# Mast Cells in Human Stenotic Aortic Valves Are Associated with the Severity of Stenosis

**DOI:** 10.1007/s10753-012-9565-z

**Published:** 2012-10-30

**Authors:** E. Wypasek, J. Natorska, G. Grudzień, G. Filip, J. Sadowski, A. Undas

**Affiliations:** 1 Institute of Cardiology, Jagiellonian University Medical College, 80 Pradnicka St, 31-202 Krakow, Poland; 2 John Paul II Hospital, Krakow, Poland

**Keywords:** aortic stenosis, mast cells, valvular calcification, transvalvular gradients

## Abstract

Aortic valve stenosis (AS) is characterized by extensive calcification of the aortic valve leaflets and infiltration of inflammatory cells. Activated mast cells (MCs) may participate in the induction of fibrosis and calcification with ensuing valve stiffening. We sought to investigate whether the number of MCs within stenotic aortic valves is associated with the severity of AS. We studied 43 patients (19 men, 24 women) with dominant AS (age, 64.2 ± 5.9 years; mean transvalvular pressure gradient, 62.11 ± 24.47 mmHg) without atherosclerotic vascular disease, undergoing elective aortic valve replacement. MCs were detected in the excised valves by immunostaining. Aortic valves from five healthy subjects obtained on autopsy served as negative controls. The number of tryptase- and chymase-positive MCs was increased in AS valves compared with the control valves (6.9 [2.3–18.9]/mm^2^ *vs.* 0.7 [0–2.2]/mm^2^, *P* = 0.0001 and 3.2 [2.1–9.4]/mm^2^ *vs.* 0.3 [0–1.9]/mm^2^, *P* = 0.002, respectively). MCs that colocalized with macrophages and neovessels were detected mainly in the calcified regions of the leaflets. The number of MCs positively correlated with maximal (*r* = 0.73, *P* < 0.0001) and mean (*r* = 0.78, *P* < 0.0001) gradients and maximal aortic jet velocity (*r* = 0.68, *P* = 0.0005). An inverse correlation with aortic valve area (*r* = −0.71, *P* = 0.0001) was also observed. Multivariate regression analysis revealed that MC number and valve thickness were significantly associated with mean transvalvular gradient (*R* ^2^ = 0.74, *P* < 0.000001) in AS patients. Increased MC number within human stenotic aortic valves is associated with the severity of AS.

## INTRODUCTION

Aortic valve stenosis (AS) is a common cardiac valve acquired defect in older subjects. The narrowing of the valve opening that occurs as a result of calcification is related to the high pressure gradient across the stenotic valve [1]. In recent years AS has been perceived as an active inflammatory process that shares many similarities with atherosclerosis, including subendothelial accumulation of oxidized lipoproteins, calcification [2], and infiltration of inflammatory cells, in particular T cells, macrophages [3], and mast cells (MCs) [4]. Upon activation, MCs are able to synthesize a large number of proinflammatory and profibrotic mediators, followed by their slow and sustained secretion [5]. In humans MCs are classified according to their protease content, with the tryptase-positive MCs expressing tryptase only and the tryptase- and chymase*-*positive MCs expressing all types of MC proteases, that is, tryptase, chymase, and MC–carboxypeptidase A [6]. MC proteases have a variety of roles, inflammatory and anti-inflammatory, protective and deleterious, in keeping with the increasingly well-appreciated contributions of mast cells in allergy, innate immunity, and tissue homeostasis [7].

Growing evidence suggests that MCs may be actively involved in the progression of AS. In stenotic aortic valves, MC-derived chymase can stimulate angiotensin II formation [4]. Increased Ang II production, in turn, may mediate collagen gene transcription by stimulating the expression of transforming growth factor-β (TGF-β) [4]. TGF-β1 was shown to be characteristically accumulated in MCs [4] as well as in the extracellular matrix and mineral deposits in calcified aortic valve cusps [2]. Studies with transgenic mice have indicated that increased mast cell density with a resultant increase in TGF-β-mediated profibrotic signaling between cardiac mast cells and resident cardiac fibroblasts is required for the development of myocardial fibrosis [8].

MCs have also been identified as a major source of cathepsin G, an enzyme with lytic actions on elastic fibers, involved in TGF-β pathway activation [9]. Furthermore, MCs located in close proximity to blood vessels together with myofibroblasts are implicated in valve neoangiogenesis [10]. These findings suggest that activated and degranulated MCs present within fibrotic lesions of the aortic valves may participate in the induction of fibrosis and calcification with ensuing valve stiffening [4, 9]. However, to the best of our knowledge, there have been no reports showing associations between MCs within stenotic aortic valves and severity of AS. Therefore, the aim of the current study was to test the hypothesis that, as in advanced atherosclerotic plaques [11], MCs may contribute to the severity of AS.

## METHODS

### Patients

A total of 43 consecutive patients (19 men and 24 women) undergoing isolated elective aortic valve replacement for moderate to severe AS were recruited. Only patients with acquired aortic stenosis were included in the study. The exclusion criteria were: a bicuspid valve, low-output low-gradient aortic stenosis and an ejection fraction <40 %, diabetes mellitus, acute infection, Valsalva sinus aneurysm or rheumatic AS, angiographically documented coronary artery stenosis >20 % diameter, carotid artery stenosis, known cancer, autoimmune disorders, endocarditis, previous cardiac surgery, a history of myocardial infarction, stroke, venous thromboembolism, or bleeding. Patients who required additional surgical intervention or had other heart defects were ineligible.

Information on the presence or absence of cardiovascular risk factors, including arterial hypertension, hyperlipidemia, smoking, diabetes mellitus, and use of statins, angiotensin-converting enzyme inhibitors (ACEIs), β-blockers, and acetylsalicylic acid, was obtained before surgery. Smoking was defined as the use of one or more cigarettes per day. Patients receiving insulin or oral hypoglycemic agents, or having at least two random fasting glucose levels of >7 mmol/l, were classified as having diabetes mellitus. Arterial hypertension was diagnosed based on a history of hypertension (blood pressure >140/90 mmHg) or preadmission antihypertensive treatment. Hyperlipidemia was diagnosed based on medical records, statin therapy, or total cholesterol of 5.2 mmol/l or more. Renal failure was diagnosed based on having at least two random fasting creatinine levels of >110 μmol/l for males and >80 μmol/l for females. Five aortic valves from age-matched healthy subjects, obtained at autopsy, without morphological AS or other valvular disorders, served as negative controls.

Patients gave their informed written consent, and the study was approved by the University Bioethical Committee.

### Echocardiography

Transthoracic echocardiography was performed in each patient using a MargotMac 5000 ultrasound machine prior to surgery using conventional techniques in accordance with the European Society of Cardiology guidelines. The aortic valve area (AVA) was calculated using the standard continuity equation [12]. The transvalvular gradient was measured by Doppler echocardiography using the modified Bernoulli equation [12].

### Laboratory Tests

Fasting venous blood was drawn from patients 24 h before surgery between 7 and 9 AM. Citrated blood samples (9:1 of 0.106 M sodium citrate) were centrifuged at 3,000 rpm at 20 °C for 10 min and stored in aliquots at −80 °C until analysis. Lipid profile, glucose, and creatinine were assayed by routine laboratory techniques. High-sensitivity C-reactive protein (CRP) was determined using immunoturbidimetry (Roche Diagnostics GmbH, Mannheim, Germany).

### Analysis of Aortic Valves

Diseased aortic valves were collected during surgery for valve replacement. After decalcification, performed as previously described [13], valves were embedded in Tissue Tec-OCT compound (Sakura, Torrance, CA, USA) for tissue cryopreservation and cryosectioned (8–10-μm-thick sections) onto SuperFrost slides (Menzel-Glaser, Germany) by a Leica Jung CM 3000 cryostat. Sections were taken transversely from the middle of the leaflet and from commissural areas. Slides were stored at −20 °C until morphological analysis and immunostaining. To ascertain the morphology of the leaflets, one hematoxylin–eosin section, taken from sections obtained from each leaflet, was observed using optical microscopy before the other cryosections were subjected to the immuno- and morphometric evaluation.

The valve thickness was measured in the lesion area, defined as total thickness of the leaflets [13].

### Immunostaining Analysis

Undecalcified serial sections were stained with von Kossa's stain (Sigma, St. Louis, MO, USA) to localize ionized calcium deposits. Immunofluorescence and immunohistochemistry were performed on adjacent sections of aortic valves as described before [13]. Briefly, after endogenous peroxidase activity quenching and blocking of unspecific background, primary adequate monoclonal antibodies were applied overnight at 4 °C against human mast cell anti-tryptase **(**Dako, Glostrup, Denmark) and anti-chymase (Serotec, Hanar, Germany), human macrophage (anti-CD68 antigen, Santa Cruz Biotechnology Inc., CA, USA), or a neovascularization marker, tyrosine kinase receptor Tie-2 (Abcam, Cambridge, UK).

Primary antibodies were followed by the corresponding secondary antibodies conjugated with fluorochrome (Santa Cruz Biotechnology Inc., CA, USA) or by the avidin–biotin complex immunoperoxidase according to the manufacturer's instructions (Santa Cruz Biotechnology Inc., CA, USA). Diaminobenzidine was used as the chromogen. A negative control (without primary antibody incubation) was included routinely. Sections were viewed in fluorescence or light microscope (both Zeiss, Berlin, Germany). Photomicrographs were taken using a Canon A640 camera and analyzed using the image analysis software CellProfiler Analyst. The exact numbers of tryptase-positive and chymase-positive MCs in the calcified region of each section were counted by light microscopy as described previously [14]. Tie-2 expression was analyzed semiquantitatively according to the following scoring system: 0, absence; 1, isolated neovessels ( < 10 % of the total leaflet area), and 2, abundant neovessels ( > 10 % of the total leaflet area).

### Valvular Calcification

Valvular calcification was analyzed semiquantitatively according to the following scoring system: 1, isolated calcium deposits; 2, calcification occupying less than 20 % of the total leaflet area, and 3, abundant calcium deposits occupying more than 20 % of the total leaflet area.

### Statistical Analysis

Values are expressed as mean (SD) or median (interquartile range) or otherwise stated. The Kolmogorov–Smirnov test was used to assess conformity with a normal distribution. Pairwise comparisons were made using Tukey's test for continuous variables and the chi^2^ test for proportions. The Mann–Whitney *U* test was used to compare non-normally distributed variables between two groups. Spearman's correlation coefficient was calculated to evaluate associations between variables. A multivariate stepwise linear regression analysis was performed to evaluate the potential contribution of clinical and demographical variables to mean transvalvular gradient. A value of *P* < 0.05 was considered statistically significant.

## RESULTS

### Laboratory Parameters

The demographic, clinical, and echocardiographic parameters in the AS patients are summarized in Table 1. Valvular stenosis was moderate to severe with maximal transvalvular gradient of 96.2 ± 25.87 mmHg and mean gradient of 62.11 ± 24.47 mmHg. Left ventricular ejection fraction was 55.8 ± 12.4 %. Plasma fibrinogen and CRP levels were 3.78 ± 0.8 g/l and 6.2 (1.22–21.2) mg/l, respectively.

**Table 1:** Patients Characteristics

	AS (*n* = 43)
Demographic data	
Male, *n* (%)	19 (44)
Age, years	64.2 ± 5.9
Body mass index (kg/m^2^)	28.3 ± 5.7
Risk factors	
Hypertension, *n* (%)	23 (53)
Hypercholesterolemia, *n* (%)	14 (33)
Currently smoking, *n* (%)	3 (7)
Medication	
Beta-blockers, *n* (%)	12 (30)
Acetylsalicylic acid, *n* (%)	23 (53)
ACEI, *n* (%)	12 (30)
Statins, *n* (%)	11 (26)
Echocardiography	
Maximum gradient, mmHg	96.2 ± 25.87
Mean gradient, mmHg	62.11 ± 24.47
LVEF, %	55.82 ± 12.42
Calcified valves, *n* (%)	43 (100)
Aortic bulb width, cm	2.68 ± 1.25 (*n* = 25)
Ascending aortic width, cm	3.63 ± 0.97
Vmax	4.82 ± 9.72 (*n* = 21)
AVA, cm^2^	0.72 ± 0.26 (*n* = 27)

Values are given as mean (SD) or number (percentage)

*ACEI* angiotensin-converting enzyme inhibitors, *AVA* aortic valve area, *LVEF* left ventricular ejection fraction, *V max* maximum aortic jet velocity

### Aortic Valve Tissue Analysis

Total valve leaflet thickness was 1.36 (0.98–2.94) mm. Focal areas of subendothelial thickening comprising roughly two thirds of the aortic side of the leaflet were seen in all AS valves as compared with the control group, in which no lesions were observed. Large amounts of calcium deposits in the fibrosa and the subendothelial layer at the aortic side of the leaflets were detected in all AS valves. Any increase in fibrosis and lipid insudation was observed in negative control valves.

### Immunohistochemistry and Immunofluorescence

**Fig. 1:**
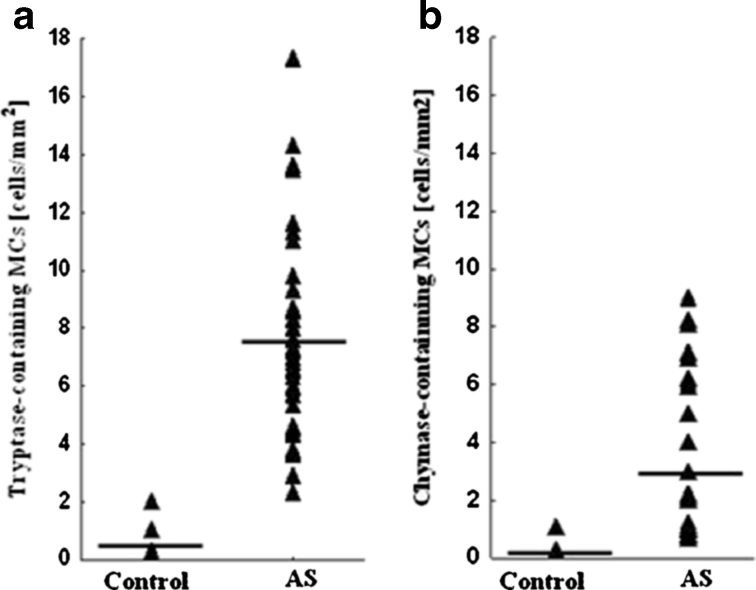
The number of tryptase- (**a**) and chymase-containing (**b**) MCs in normal (*control*) and stenotic (*AS*) aortic valves. *Lines* indicate means.

**Fig. 2:**
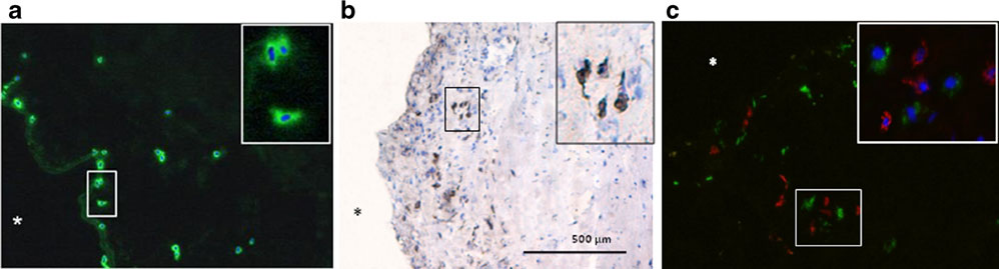
Representative micrographs of MC staining within stenotic aortic valves using immunofluorescence (**a**, **c**; *green*) and immunohistochemistry (**b**, *brown*). Double-labeled immunostaining (**c**) shows that MCs (*green*) are present in the vicinity of macrophages (*red*). Nuclei were counterstained with DAPI (*blue*). Original magnification, ×40; insert, ×100. *aortic side of the leaflet.

AS valves were characterized by an increased number of tryptase- and chymase-positive MCs, compared with the control valves (6.9 [2.3–18.9]/mm^2^ *vs.* 0.7 [0–2.2]/mm^2^, *P* = 0.0001, and 3.2 [2.1–9.4]/mm^2^ *vs.* 0.3 [0–1.9]/mm^2^, *P* = 0.002, respectively) (Fig. 1). MCs were noted in 100 % of the stenotic valves. The accumulation of MCs was evident in the subendothelial region as well as throughout the leaflets predominantly in the calcified areas (Fig. 2).

The regions of the aortic valve showing abundant calcification had increased MC numbers compared with those with isolated calcium deposits (8.82 ± 3.31/mm^2^ *vs.* 4.82 ± 0.94/mm^2^, *P* = 0.0003). Moreover, there was a significant difference in the degree of calcification in patients with mean transvalvular gradient more than 50 mmHg compared to those with a gradient less than or equal to 50 mmHg (2.68 ± 0.15 *vs.* 2.25 ± 0.16/mm^2^, *P* = 0.05). Larger Tie-2-positive areas were associated with a higher number of MCs compared to those without Tie-2 expression (8.84 ± 2.02/mm^2^ *vs.* 5.06 ± 1.14/mm^2^, *P* = 0.0003). Any correlation between MC numbers and valve thickness was observed.

MCs colocalized with macrophages mainly in the lesion area of the stenotic aortic valves (Fig. 2c). There was a strong positive correlation between the number of MCs and macrophages (*r* = 0.66, *P* = 0.0001). Quartile analysis of plasma CRP levels showed that AS patients in the highest quartile (>3.90 mg/l, *n* = 11) had elevated numbers of MC compared to those in the lowest (<1.20 mg/l, *n* = 11) (8.62 ± 3.41/mm^2^ *vs.* 6.13 ± 1.83/mm^2^, *P* = 0.03).

**Fig. 3:**
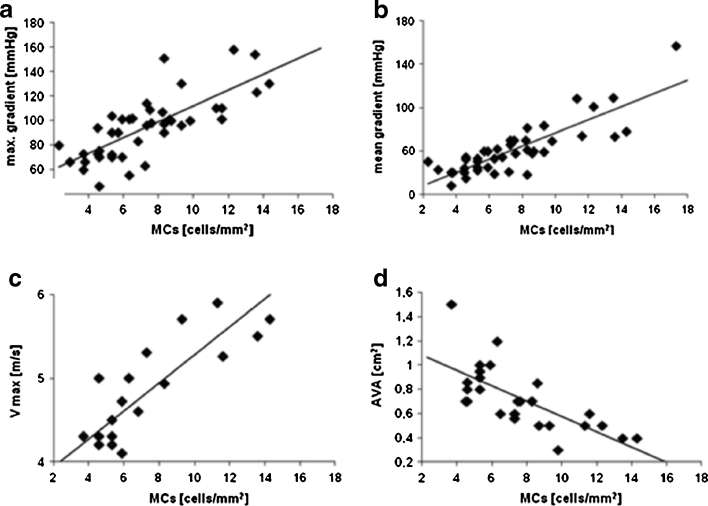
Correlation between transvalvular maximal gradient (**a**), mean gradient (**b**), maximum aortic jet velocity (*Vmax*, **c**), aortic valve area (*AVA*, **d**), and MC expression.

**Fig. 4:**
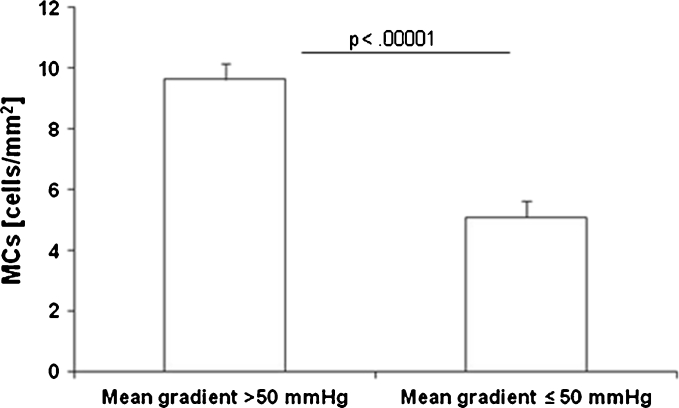
Associations between MC expression and mean transvalvular gradient in AS patients with gradient more than 50 mmHg or less than or equal to 50 mmHg. Values are given as mean ± standard deviation.

The number of MCs was positively correlated with maximal (*r* = 0.73, *P* < 0.0001) and mean (*r* = 0.78, *P* < 0.0001) gradients and maximal aortic jet velocity (*r* = 0.68, *P* = 0.0005), while an inverse correlation of the number of MCs with AVA (*r* = −0.71, *P* < 0.0001) was observed (Fig. 3). Patients with severe AS with a mean gradient of >50 mmHg had higher MC numbers than those with mean gradient ≤50 mmHg (9.64 ± 0.51 *vs.* 5.10 ± 0.53/mm^2^, *P* < 0.0001) (Fig. 4).

There were no associations between demographic variables or cardiovascular risk factors and valvular MC numbers in AS patients (data not shown). A multivariate stepwise linear regression analysis identified the MC numbers and valve thickness as the independent predictors of mean transvalvular gradient (*R* ^2^ = 0.74, *P* < 0.000001) in AS patients (Table 2).

**Table 2:** Summary of the Additive Regression Models (Stepwise Method): Predictor Contribution to the Mean Transvalvular Gradient in AS Patients

*R* ^2^ = 0.74, *P* < 0.00001	β coefficient	SE	*P* value
MC (cells/mm^2^)	6.05	0.59	<0.00001
Valve thickness	8.36	3.34	0.02

## DISCUSSION

We demonstrate that increased MC numbers within human stenotic aortic valves, mainly in calcified areas, are associated with the severity of AS. This study is the first to show that the higher the number of MCs, the more severe AS is in subjects without evidence of atherosclerotic vascular disease. The presence of numerous degranulated MCs and their local accumulation has been described within human aortic valves by Helske et al. [4]. MCs can also be detected from the earliest to the most advanced stages of atherosclerosis [15], and their number increased linearly with increasing severity of the disease [16]. During atherosclerotic lesion progression, it has been shown that MCs accumulate in the rupture-prone shoulder region of human atheromas [11] and in the perivascular tissue (adventitia) reaching even 104 ± 15 cells/mm^2^ in the most advanced lesions [16]. Our findings indicate that in AS patients showing no evidence of coronary or carotid stenosis, significant amounts of MCs, though lower than in unstable plaques, are present within their valves in association with transvalvular gradients and AVA.

It has been demonstrated that accumulation of MCs within inflamed tissue could occur by local proliferation of resident MCs, increased recruitment of MC precursors from the circulation, or migration of mature MCs from adjacent tissues. In our study, MCs were found in calcified areas and in the subendothelial layer on the aortic side of the stenotic leaflets, which is consistent with previous findings [4, 9]. The aortic side of the leaflets is an area of flow perturbations and low shear stress, so predisposing to endothelial injury and subsequent infiltration of inflammatory cells and plasma lipoproteins [3]. We may speculate that the damaged side of the leaflets may be the region of MC migration from circulation to the inflammatory lesion. Undoubtedly, macrophages accumulating in large numbers within stenotic aortic valves [3] are key players in the pathogenesis of AS. They secrete a variety of factors that activate valve myofibroblasts and trigger their osteoblastic transdifferentiation as well as promote monocyte migration into developing aortic valve stenosis [17]. MCs may be a secondary phenomenon to degeneration and calcification of aortic leaflets. Our immunohistochemical analysis revealed colocalization of MCs with macrophages around the calcified areas, suggesting their close cooperation. Moreover, similarly to atherosclerotic plaques, activated MCs may contribute to macrophage accumulation within valvular lesions either by their direct recruitment [18] or by inducing the secretion of monocyte chemoattractant protein-1 from fibroblasts [19].

We have also found that the MC numbers were associated with the expression of Tie-2 marker suggesting that neovascularization enhances infiltration of MCs and/or *vice versa*. It has been reported that in AS valves, MCs actively contribute to valve neoangiogenesis as they stored and secreted proangiogenic vascular endothelial growth factor [10]. The presence of neovessels may facilitate the influx of substances implicated in aortic valve degeneration and increase macrophage recruitment [10].

Interestingly, during persistent inflammation and tissue remodeling, MC populations can undergo significant changes, not only in the number but also in the content of stored mediators, in response to microenvironmental changes [20]. In atherosclerotic lesions activated MCs promote atherosclerotic plaque progression and destabilization by increasing the risk of intraplaque hemorrhage, intimal leukocyte influx, and macrophage apoptosis [21]. As in atherosclerosis, the final result is plaque development and plaque instability whereas in AS, severe calcification of the aortic valve and fibrocalcific thickening is the end stage of the disease. We have confirmed that the degree of valvular calcification in AS valves corresponds with an increased number of MCs. Moreover, in our study severe leaflet calcification was associated with higher transvalvular gradient.

It is known that MCs participate in the pathogenesis of aortic valve calcification by secreting the proteases tryptase, chymase, and cathepsin G, which may directly degrade the extracellular matrix of valves [4, 9]. Mast cell-derived chymase, in addition to forming Ang II, may contribute to valvular fibrosis by promoting MC migration and degranulation and by generating an active form of TGF-β [5]. Within aortic valves, TGF-β enhances collagen synthesis and may contribute to the progression of aortic stenosis by initiating the apoptosis-related calcification of aortic valve interstitial cells [2]. The presence of activated MCs in the inflamed stenotic valves and their contribution to severe leaflet calcification and mineralization may lead to increased transvalvular gradients and decreased aortic valve area. Our findings confirm this hypothesis as we observed strong positive associations between MC numbers and maximal and mean gradient as well as maximum aortic jet velocity. Moreover, valvular MC numbers were significantly increased in patients with severe AS compared to those with moderate AS. Importantly, we have indicated that MC numbers are the best predictors of mean transvalvular gradient which supports the hypothesis that the presence of MCs contributes to the progression of AS.

In our study increased MC numbers was also associated with elevated serum CRP levels which may reflect chronic inflammation in diseased aortic valves and activity of inflammatory cells. Indeed it was shown by others that ongoing inflammation in the valve leaflets is associated with elevated serum CRP level [22]. One might speculate that CRP influences MC number through complement activation which occurs in the stenotic valve leaflets [23]. C3a and C5a have been shown to participate in MC recruitment [24] and are able to induce their activation. On the other hand it is also possible that CRP itself may participate in valve calcification. In an *in vitro* simulating model, rising CRP levels progressively increased the rate of aortic wall calcification [25].

The study has several limitations. First, the number of the patients enrolled in this study was small. Secondly, our findings likely cannot be extrapolated on subjects with mild AS or with aortic sclerosis as we focused on the valves obtained from patients with severe AS scheduled for surgery. Third, we did not perform double staining to distinguish chymase- and tryptase-positive cells from tryptase-positive cells to determine which subtypes more strongly determine AS severity.

In summary, we showed that MCs are associated with the severity of AS in humans, suggesting an active involvement of these cells in the progression of this disease. Increased MC numbers present in human AS valves enhance valvular inflammation associated with aortic valve calcification and neovascularization, restricted leaflet motion, and AS severity. It is tempting to hypothesize that inhibition of MC activation and degranulation within stenotic aortic valves may be an important target in the development of therapeutic approaches to prevent progression of AS. This issue merits further investigation.

## References

[1] Olszowska M (2011). Pathogenesis and pathophysiology of aortic valve stenosis in adults. Polskie Archiwum Medycyny Wewnętrznej.

[2] Jian B, Narula N, Li QY, Mohler ER, Levy RJ (2003). Progression of aortic valve stenosis: TGF-beta1 is present in calcified aortic valve cusps and promotes aortic valve interstitial cell calcification via apoptosis. The Annals of Thoracic Surgery.

[3] Otto CM, Kuusisto J, Reichenbach DD, Gown AM, O’Brien KD (1994). Characterization of the early lesion of ‘degenerative’ valvular aortic stenosis. Histological and immunohistochemical studies. Circulation.

[4] Helske S, Lindstedt KA, Laine M, Mäyränpää M, Werkkala K, Lommi J, Turto H, Kupari M (2004). Induction of local angiotensin II-producing systems in stenotic aortic valves. Journal of the American College of Cardiology.

[5] Lindstedt KA, Wang Y, Shiota N, Saarinen J, Hyytiäinen M, Kokkonen JO, Keski-Oja J, Kovanen PT (2001). Activation of paracrine TGF-beta1 signaling upon stimulation and degranulation of rat serosal mast cells: a novel function for chymase. The FASEB Journal.

[6] Pejler G, Rönnberg E, Waern I, Wernersson S (2010). Mast cell proteases: multifaceted regulators of inflammatory disease. Blood.

[7] Caughey GH (2011). Mast cell proteases as protective and inflammatory mediators. Advances in Experimental Medicine and Biology.

[8] Zhang W, Chancey AL, Tzeng HP, Zhou Z, Lavine KJ, Gao F, Sivasubramanian N, Barger PM, Mann DL (2011). The development of myocardial fibrosis in transgenic mice with targeted overexpression of tumor necrosis factor requires mast cell-fibroblast interactions. Circulation.

[9] Helske S, Syväranta S, Kupari M, Lappalainen J, Laine M, Lommi J, Turto H, Mäyränpää M, Werkkala K, Kovanen PT, Lindstedt KA (2006). Possible role for mast cell-derived cathepsin G in the adverse remodelling of stenotic aortic valves. European Heart Journal.

[10] Syväranta S, Helske S, Laine M, Lappalainen J, Kupari M, Mäyränpää MI, Lindstedt KA, Kovanen PT (2010). Vascular endothelial growth factor-secreting mast cells and myofibroblasts: a novel self-perpetuating angiogenic pathway in aortic valve stenosis. Arteriosclerosis, Thrombosis, and Vascular Biology.

[11] Kovanen PT, Kaartinen M, Paavonen T (1995). Infiltrates of activated mast cells at the site of coronary atheromatous erosion or rupture in myocardial infarction. Circulation.

[12] Teirstein P, Yeager M, Yock PG, Popp RL (1986). Doppler echocardiographic measurement of aortic valve area in aortic stenosis: a noninvasive application of the Gorlin formula. Journal of the American College of Cardiology.

[13] Natorska J, Marek G, Hlawaty M, Sadowski J, Tracz W, Undas A (2011). Fibrin presence within aortic valves in patients with aortic stenosis: association with *in vivo* thrombin generation and fibrin clot properties. Thrombosis and Haemostasis.

[14] Lamprecht MR, Sabatini DM, Carpenter AE (2007). Cell Profiler: versatile software for automated biological image analysis. Biotechniques.

[15] Jeziorska M, McCollum C, Woolley DE (1997). Mast cell distribution, activation, and phenotype in atherosclerotic lesions of human carotid arteries. The Journal of Pathology.

[16] Laine P, Naukkarinen A, Heikkilä L, Penttilä A, Kovanen PT (2000). Adventitial mast cells connect with sensory nerve fibers in atherosclerotic coronary arteries. Circulation.

[17] Joghetaei N, Akhyari P, Rauch BH, Cullen P, Lichtenberg A, Rudelius M, Pelisek J, Schmidt R (2011). Extracellular matrix metalloproteinase inducer (CD147) and membrane type 1-matrix metalloproteinase are expressed on tissue macrophages in calcific aortic stenosis and induce transmigration in an artificial valve model. The Journal of Thoracic and Cardiovascular Surgery.

[18] Tani K, Ogushi F, Kido H, Kawano T, Kunori Y, Kamimura T, Cui P, Sone S (2000). Chymase is a potent chemoattractant for human monocytes and neutrophils. Journal of Leukocyte Biology.

[19] Gordon JR (2000). TGFbeta1 and TNFalpha secreted by mast cells stimulated via the FcepsilonRI activate fibroblasts for high-level production of monocyte chemoattractant protein-1 (MCP-1). Cellular Immunology.

[20] Galli SJ, Tsai M (2010). Mast cells in allergy and infection: versatile effector and regulatory cells in innate and adaptive immunity. European Journal of Immunology.

[21] Bot I, de Jager SC, Zernecke A, Lindstedt KA, van Berkel TJ, Weber C, Biessen EA (2007). Perivascular mast cells promote atherogenesis and induce plaque destabilization in apoE deficient mice. Circulation.

[22] Galante A, Pietroiusti A, Vellini M, Piccolo P, Possati G, De Bonis M, Grillo RL, Fontana C, Favalli C (2001). C-reactive protein is increased in patients with degenerative aortic valvular stenosis. Journal of the American College of Cardiology.

[23] Helske S, Oksjoki R, Lindstedt KA, Lommi J, Turto H, Werkkala K, Kupari M, Kovanen PT (2008). Complement system is activated in stenotic aortic valves. Atherosclerosis.

[24] Wojta J, Kaun C, Zorn G, Ghannadan M, Hauswirth AW, Sperr WR, Fritsch G, Printz D, Binder BR, Schatzl G, Zwirner J, Maurer G, Huber K, Valent P (2002). C5a stimulates production of plasminogen activator inhibitor-1 in human mast cells and basophils. Blood.

[25] Warrier B, Mallipeddi R, Karla PK, Lee CH (2005). The functional role of C-reactive protein in aortic wall calcification. Cardiology.

